# Linkage between the intestinal microbiota and residual feed intake in broiler chickens

**DOI:** 10.1186/s40104-020-00542-2

**Published:** 2021-02-11

**Authors:** Jing Liu, Sydney N. Stewart, Kelsy Robinson, Qing Yang, Wentao Lyu, Melanie A. Whitmore, Guolong Zhang

**Affiliations:** 1 Department of Animal and Food Sciences, Oklahoma State University, Stillwater, OK USA; 2 Present address: Poultry Production and Product Safety Research Unit, USDA-Agricultural Research Service (ARS), Fayetteville, AR USA; 3 Institute of Quality and Standards for Agro-products, Zhejiang Academy of Agricultural Sciences, Hangzhou, China

**Keywords:** Feed conversion ratio, Feed efficiency, Microbiota, Poultry, Residual feed intake

## Abstract

**Background:**

Intestinal microbiota plays a key role in nutrient digestion and utilization with a profound impact on feed efficiency of livestock animals. However, the intestinal microbes that are critically involved in feed efficiency remain elusive.

**Methods:**

To identify intestinal bacteria associated with residual feed intake (RFI) in chickens, male Cobb broiler chicks were individually housed from day 14 to day 35. Individual RFI values were calculated for 56 chickens. Luminal contents were collected from the ileum, cecum, and cloaca of each animal on day 35. Bacterial DNA was isolated and subjected to 16S rRNA gene sequencing. Intestinal microbiota was classified to the feature level using Deblur and QIIME 2. High and low RFI groups were formed by selecting 15 and 17 chickens with the most extreme RFI values for subsequent LEfSe comparison of the difference in the microbiota. Spearman correlation analysis was further performed to identify correlations between the intestinal microbiota composition and RFI.

**Results:**

No significant difference in evenness, richness, and overall diversity of the microbiota in the ileum, cecum, or cloaca was observed between high and low RFI chickens. However, LEfSe analysis revealed a number of bacterial features being differentially enriched in either high or low RFI chickens. Spearman correlation analysis further identified many differentially enriched bacterial features to be significantly correlated with RFI (*P* < 0.05). Importantly, not all short-chain fatty acid (SCFA) producers showed a positive association with RFI. While two novel members of *Oscillibacter* and *Butyricicoccus* were more abundant in low-RFI, high-efficiency chickens, several other SCFA producers such as *Subdoligranulum variabile* and two related Peptostreptococcaceae members were negatively associated with feed efficiency. Moreover, a few closely-related Lachnospiraceae family members showed a positive correlation with feed efficiency, while others of the same family displayed an opposite relationship.

**Conclusions:**

Our results highlight the complexity of the intestinal microbiota and a need to differentiate the bacteria to the species, subspecies, and even strain levels in order to reveal their true association with feed efficiency. Identification of RFI-associated bacteria provides important leads to manipulate the intestinal microbiota for improving production efficiency, profitability, and sustainability of poultry production.

## Introduction

Feed accounts for up to 70% of the costs in broiler production [1]. Maximizing feed efficiency is paramount to ensuring the profitability and sustainability of the industry. In livestock production, feed efficiency is generally measured by feed conversion ratio (FCR) or residual feed intake (RFI). FCR is defined as the ratio of feed intake to weight gain, with lower FCR values indicating higher efficiency. On the other hand, RFI is defined as the difference between actual measured feed intake and expected feed intake of an animal accounting for its maintenance requirement, where expected feed intake is calculated based on average feed intake and weight grain of a group of animals [2, 3]. Similar to FCR, a lower RFI value indicates higher efficiency. However, unlike FCR, which measures the ratio of two biological traits (feed intake and growth rate), RFI measures feed efficiency independent of body weight, mature size, or growth rate [2, 4]. A difference in RFI among animals is most likely due to a variation in the maintenance energy expenditure. Long-term selection of animals based on FCR often leads to larger animals that consume more feed, while RFI selection results in comparable animal sizes and production levels with reduced feed intake [2–4]. RFI is, therefore, becoming a method of choice for measuring feed efficiency [2–4].

Intestinal microbiota is known to play a key role in nutrient digestion and absorption, vitamin synthesis, and immune development [5–8]. Manipulation of the intestinal microbiota could potentially enhance animal health and feed efficiency [8]. Relative to that of other livestock species, the chicken intestinal microbiota has a higher proportion of Firmicutes to Bacteroidetes [9]. Lactobacilli are predominant in the small intestine, while clostridia abundantly colonize the cecum of chickens [8, 9]. As major producers of short-chain fatty acids (SCFAs), clostridia are represented by a large diverse group of obligate anaerobic Firmicutes [10, 11]. Several clostridial families such as Clostridiaceae*,* Ruminococcaceae, and Lachnospiraceae are generally regarded to improve feed efficiency through SCFA production [12–14].

Microbiome studies of broiler chickens have revealed a high degree of inter-flock variation, with diet, environment, management, age, and breed exerting a significant influence on the composition and function of the intestinal microbiome [15–17]. Several studies have attempted to identify the intestinal microbes associated with RFI in both broiler and layer chickens [18–23]. However, the findings thus far have been inconsistent and sometimes contradictory. No specific bacterial taxa have been reproducibly identified across studies. Even the same dietary composition and experimental design being duplicated at different locations have resulted in different outcomes [19].

Moreover, all aforementioned studies have classified bacteria to the level of genus or operational taxonomic unit (OTU), which arbitrarily combines all sequencing reads that share ≥97% identity as a single unit [24]. In those studies, only differentially enriched bacterial genera or OTUs were identified [18–23]. A need, therefore, exists to clarify the discrepancies among these studies and potentially further identify specific RFI-associated microbes to the species or subspecies level. Deblur, a newly-developed bioinformatic tool, separates rather than combines closely-related bacterial taxa even with a single nucleotide difference [25]. Each unique sequence is referred to as an amplicon sequence variant (ASV) or a ‘feature’. It is, therefore, now possible to accurately define the microbiota composition and compare them among studies [25, 26].

In this study, to identify bacterial features that are associated with feed efficiency, we housed broilers individually, calculated their RFI values, analyzed the compositions of the microbiome in the ileum, cecum, and cloaca separately using Deblur, and further compared them between two groups of broilers with extremely high and low RFI values. As a result, a number of bacterial features were found to be differentially enriched between high and low RFI broilers in each of the three intestinal locations. We were able to separate closely-related bacteria from each other and we found, in several cases, both positive and negative associations with feed efficiency among them, highlighting a need to differentiate phylogenetically related bacteria from each other in order to reveal their true involvement in nutrient digestion and utilization and possibly other physiological processes, particularly when microbiome differences are subtle.

## Materials and methods

### Animal trial and sample collection

All animal procedures were approved by the Institutional Animal Care and Use Committee of Oklahoma State University under protocol number AG-17-3. A total of 400 day-of-hatch male Cobb broiler chicks were obtained from Cobb-Vantress Hatchery (Siloam Springs, AR). Upon arrival, chicks were individually weighed and apparently healthy animals with similar body weight (BW) (40 ± 2 g) were retained. Animals were tagged with wing bands and group-housed on an open floor with fresh pinewood shavings for bedding. Chickens were provided *ad libitum* access to tap water and non-medicated, three-stage, corn-soybean diets formulated to meet or exceed the NRC requirements (Table S1). Animals were housed in an environmentally-controlled room with temperatures starting at 33 °C and decreasing 2–3 °C every 7 days till it reaches 19 °C. The light-to-dark ratio (h:h) was 24:0 for day 0, 23:1 for days 1 to 3, 18:6 for days 4 to 8, and 16:8 for days 9 to 35. On day 14, all animals were individually weighed and 72 apparently healthy chickens were selected to reflect the body weight range of the entire group of animals and transferred to individual floor cages with fresh pinewood bedding. From day 14 to day 35, BW and feed intake were recorded individually on a weekly basis.

On day 35, all apparently healthy chickens were euthanized by CO_2_ asphyxiation, followed by cervical dislocation. Approximately 0.2–0.5 g of the ileal and cecal contents as well as 0.1 g of the cloacal content were collected from each bird and flash frozen in liquid nitrogen. The samples were stored at − 80 °C until further processing. Feed efficiency of individual animals was calculated as RFI = TFI – (a_1_ + b_1_ *×* MMW + b_2_ × TBWG), where TFI is total feed intake, a_1_ is the intercept, b_1_ and b_2_ are partial regression coefficients of mid-test metabolic weight (MMW), TBWG is total body weight gain, and MMW was calculated as [(BW_D14_ + BW_D35_)/2]^0.75^ as described [27].

### Bacterial DNA extraction and sequencing

Intestinal bacterial DNA was isolated from each luminal content sample using ZR Fecal 96-well DNA Isolation Kit (Zymo Research, Irvine, CA, USA) according to the manufacturer’s instructions. DNA quality and quantity were determined using Nanodrop ND-1000 (NanoDrop Technologies, Wilmington, DE, USA), and the absence of degradation was confirmed using agarose gel electrophoresis. High quality DNA was shipped on dry ice to Novogene (Beijing, China) for PE250 deep sequencing of the 16S rRNA gene using the V3-V4 primers (341F: CCTAYGGGRBGCASCAG and 806R: GGACTACNNGGGTATCTAAT) on an Illumina HiSeq platform. PCR amplification and library preparation were performed by Novogene (Beijing, China) using NEBNext® Ultra™ Library Prep Kit (New England Biolabs, Ipswich, MA, USA).

### Bioinformatic and statistical analyses

Illumina paired-end sequencing reads were analyzed in QIIME 22019.7 [28]. Briefly, primers were removed from each read with the cut-adapt plugin (v. 2.10) [29]. After quality filtering, reads were trimmed to 403 nucleotides and denoised with the Deblur algorithm (v. 2020.2.0). The resulting ASVs were then classified into bacterial features using the RDP 16S rRNA training set (v. 16) and Bayesian classifier [30]. A bootstrap confidence of 80% was used for taxonomic classification. Features with a classification of < 80% were assigned the name of the last confidently assigned level followed by “_unidentified”. Features appearing in < 5% of samples were removed from analysis. Data were normalized using cumulative sum scaling (CSS) in the metagenomeSeq package of R (v. 3.6.3) [31].

Analysis and visualization of the microbiota composition were conducted in R (v. 3.6.3) [32]. The α- and β-diversity were calculated with the phyloseq package (v. 1.28.0), while plots were made using ggplot2 (v. 3.3.0). The α-diversity was calculated using number of features, Shannon Index, and Pielou’s Evenness Index. The β-diversity was calculated using Bray-Curtis and Jaccard indices. Statistical significance in α-diversity and relative abundance were determined using non-parametric Kruskal-Wallis test in R. Significance in β-diversity was determined using non-parametric permutational multivariate analysis of variance (PERMANOVA) via the adonis function in the vegan package (v. 2.5-6) [33].

Differential enrichment of bacterial features between high and low RFI chickens was determined using linear discriminant analysis (LDA) effect size (LEfSe) [34], with the all-against-all multiclass analysis, *P* < 0.05, and a logarithmic LDA threshold of 2.0. Spearman correlation analysis was performed on all bacterial features in the ileum, cecum, and cloaca of all 56 chickens using the psych package (v. 1.9.12.31). Associations were considered significant if *P* ≤ 0.05 and |R| ≥ 0.3. To minimize type I error, rare bacterial features with average relative abundances of < 0.01% in an intestinal segment were excluded in both LEfSe and Spearman correlation analyses. BLAST search of the GenBank database was further conducted to reveal the identities of those features showing differential enrichment or significant correlations with RFI. Multiple sequence alignment was conducted and sequence percent identities were revealed using Clustal Omega at https://www.ebi.ac.uk/Tools/msa/clustalo/.

### Data deposition

Raw sequencing reads of this study was deposited in the NCBI GenBank SRA database under the accession number PRJNA647670.

## Results

### Production parameters

**Fig. 1:**
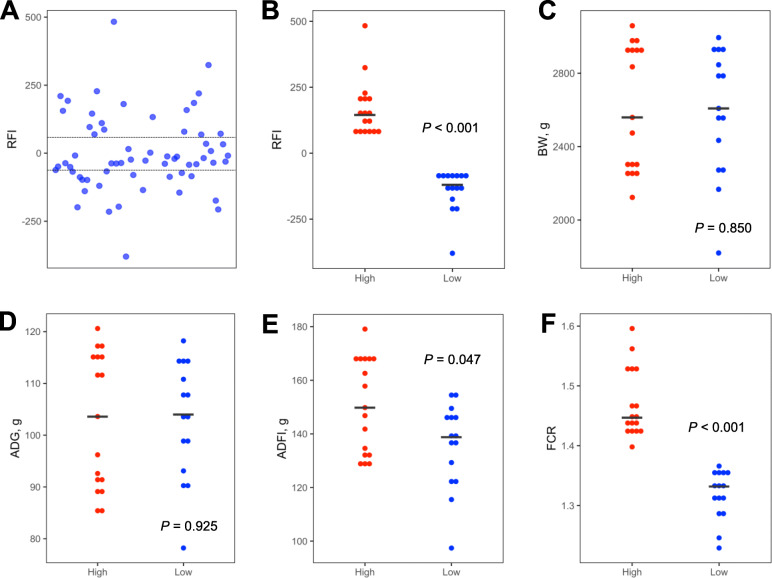
Production performance of the chickens with extremely high and low RFI values. Male Cobb chickens were individually housed from day 14 to 35 with free access to non-medicated feed. Residual feed intake (RFI) was calculated individually for 56 apparently healthy chickens, from which 15 and 17 chickens with extremely high and low RFI values, respectively, were selected (as shown by dashed lines) (**a**). RFI (**b**), body weight (BW) (**c**), average daily gain (ADG) (**d**), average daily feed intake (ADFI) (**e**), and feed conversion ratio (FCR) (**f**) were calculated for two groups of selected chickens. Statistical significance was determined using Student’s t-test

From a group of 72 individually housed broiler chickens, we were able to isolate high-quality bacterial DNA samples from the ileum, cecum, and cloaca of 56 apparently healthy chickens on day 35 for 16S rRNA gene sequencing. Sixteen animals were excluded because of subtle health issues or our inability to extract a sufficient quantity or quality of bacterial DNA for sequencing. Those 56 chickens that were retained displayed a large variation in feed efficiency with the RFI values ranging from − 379.9 to 483.1 (Fig. 1a). We selected 15 and 17 chickens with the most extreme RFI values to form ‘high’ and ‘low’ RFI groups for comparison (Fig. 1a). These two groups of chickens indeed showed a significant difference in RFI (*P* < 0.001) with an average RFI value of 166.5 and − 141.7 for high and low groups, respectively (Fig. 1b). As expected, there was no difference in day-35 BW (Fig. 1c) or average daily gain (ADG) (Fig. 1d), but significant differences in average daily feed intake (ADFI) (*P* = 0.047) (Fig. 1e) and feed conversation ratio (FCR) (*P* < 0.001) (Fig. 1f) were observed between high and low RFI groups.

### Diversity of the intestinal microbiome

**Fig. 2:**
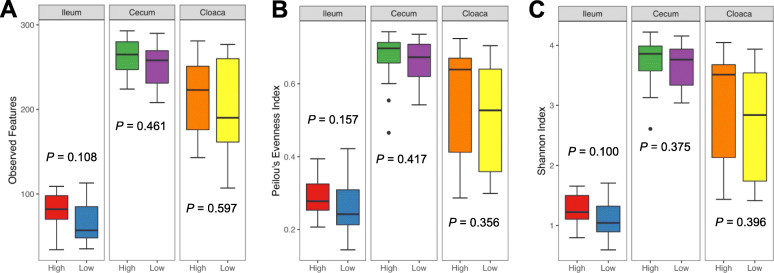
Alpha diversity of the ileal, cecal, and cloacal microbiota on day 35 between high and low RFI chickens. Differences in richness, overall diversity, and evenness were calculated using observed features (**a**), Pielou’s Evenness Index (**b**), and Shannon Index (**c**), respectively. Results were plotted using box and whisker plots, in which the middle line denoted the median value and the lower and upper hinges represented the first and third quartiles, respectively. Whiskers extended from the hinge to the highest or lowest value no farther than 1.5 × the interquartile range. Points outside of this range are considered outliers. Statistical significance was determined using Kruskal-Wallis test

**Fig. 3:**
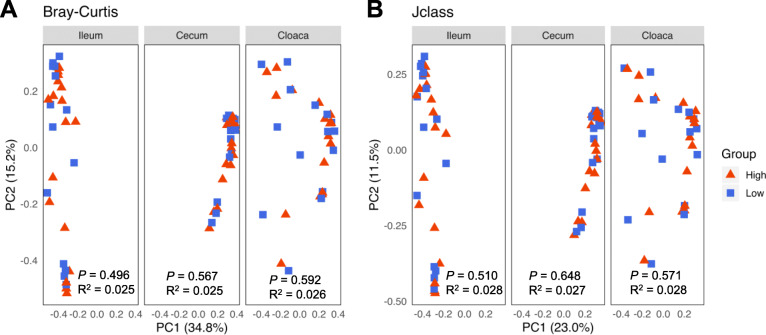
Beta diversity of the ileal, cecal, and cloacal microbiota on day 35 between high and low RFI chickens. Principal coordinates analysis (PCoA) plots were generated using Bray-Curtis (**a**) and Jaccard indices (**b**), respectively. Statistical significance and R-values were determined using permutational multivariate analysis of variance (PERMANOVA)

A total of 166 luminal content samples of the day-35 ileum, cecum, and cloaca were subjected to 16S rRNA gene sequencing. Following quality control, 11,027,919 high-quality sequencing reads were obtained with an average of 66433 ± 7117 sequences per sample. Sequences were further denoised by Deblur, and the reads present in < 5% of samples were removed, resulting in a total of 551 bacterial features. The α-diversity of the intestinal microbiota was calculated using observed features, Pielou’s Evenness Index, and Shannon Index as indications of richness, evenness, and overall diversity, respectively. No significant difference was observed in the ileum, cecum, or cloaca with any of these three indices between high and low RFI chickens (Fig. 2). The β-diversity was further calculated using the Bray-Curtis and Jaccard indices as indications of dissimilarity in overall diversity and richness, respectively. No significant separation between high and low RFI groups was observed in any of the intestinal locations (Fig. 3).

### Composition of the intestinal microbiome

**Fig. 4:**
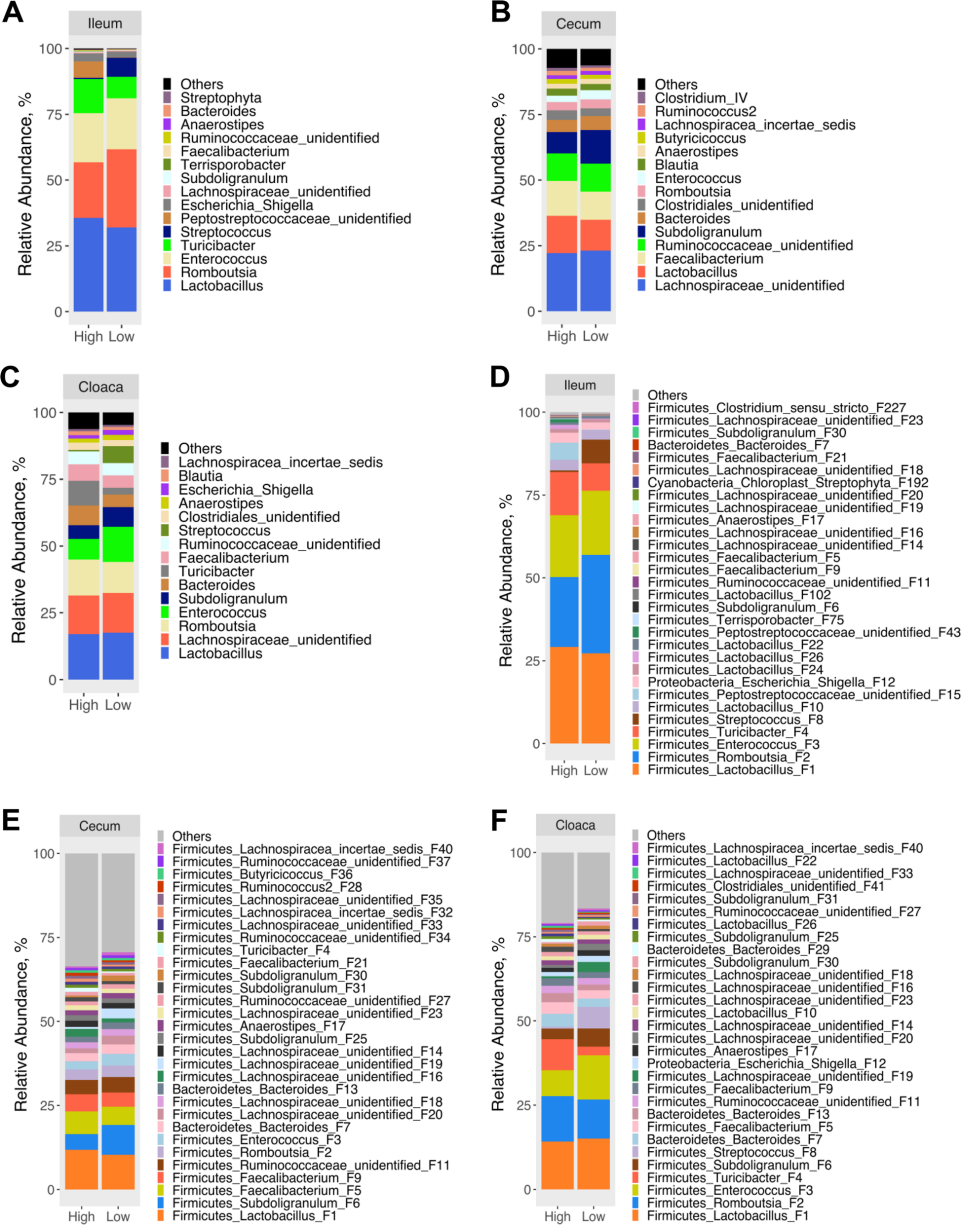
Composition of the ileal, cecal, and cloacal microbiota on day 35 between high and low RFI chickens. Relative abundance of the top 15 genera (**a**, **b**, and **c**) and top 30 features (**d**, **e**, and **f**) were shown at each intestinal location

The compositions of the microbiota were apparently different among the ileum, cecum, and cloaca. At the genus level, the ileal microbiota was dominated by *Lactobacillus, Romboutsia, Enterococcus*, and *Turicibacter* (Fig. 4a), while *Lactobacillus, Faecalibacterium,* and an unidentified genus in each of the Lachnospiraceae and Ruminococcaceae families dominated the cecal microbiota (Fig. 4b). On the other hand, the four most predominant genera of the cloacal microbiota included *Lactobacillus, Romboutsia, Enterococcus*, and an unidentified genus of the Lachnospiraceae family (Fig. 4c). However, statistical analysis of the top 15 most abundant genera in each of the ileum, cecum and cloaca revealed no significant difference (FDR > 0.05), although *Subdoligranulum* (*P* = 0.020), *Anaerostipes* (*P* = 0.027), and an unidentified genus in Peptostreptococcaceae (*P* = 0.013) and an unidentified genus in Ruminococcaceae (*P* = 0.030) tended to be more abundant in the ileum of high RFI chickens (Table S2).

At the feature level, four bacterial features including *Lactobacillus* F1*, Romboutsia* F2*, Enterococcus* F3, and *Turicibacter* F4 accounted for > 80% of the bacterial population in the ileum (Fig. 4d), while the microbiota was much more diverse in the cecum with top 30 features totaling < 70% of the bacteria (Fig. 4e). In the cloaca, four most abundant features, i.e., *Lactobacillus* F1*, Romboutsia* F2*, Enterococcus* F3, and *Turicibacter* F4, accounted for < 45% bacteria, with top 30 features totaling approximately 80% (Fig. 4f). Statistical analysis of the top 30 features revealed no significant difference (FDR > 0.05) between high and low RFI chickens in the ileum, cecum, or cloaca, while *Subdoligranulum* F6 (*P* = 0.003), *Anaerostipes* F17 (*P* = 0.015), and two unidentified features of Peptostreptococcaceae F15 (*P* = 0.018) and F43 (*P* = 0.009) tended to be more abundant in the ileum of high RFI chickens (Table S3). While there was no tendency for any feature to show differential enrichment in the cecum of high RFI chickens, an unidentified Lachnospiraceae F33 tended to be more abundant (*P* = 0.002) in the cloaca of high RFI chickens. No feature in the cecum showed statistical difference (*P* > 0.05) between high and low RFI chickens (Table S3).

### Differential enrichment of the intestinal microbiome

**Fig. 5:**
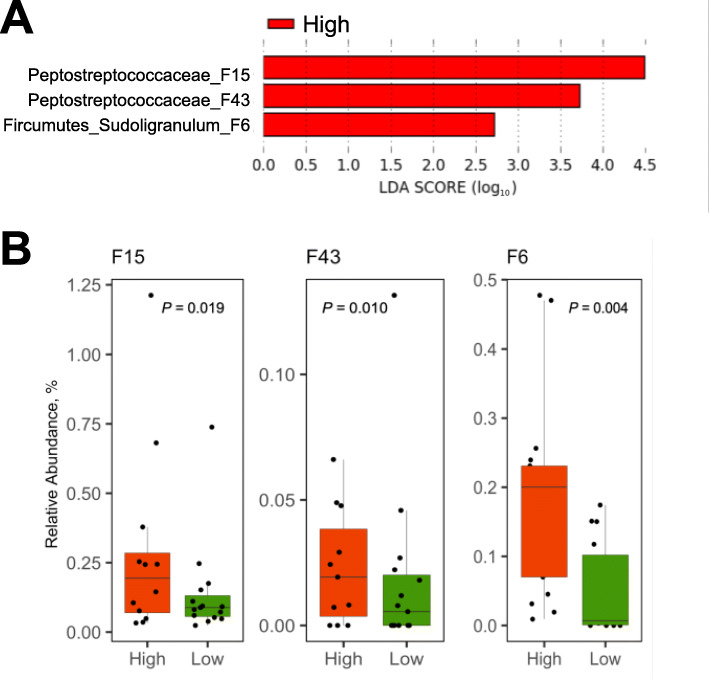
LEfSe analysis of the day-35 ileal microbiota of high and low RFI chickens. **a** Differential enrichment of the bacterial features was determined using LEfSe with a logarithmic LDA threshold of 2.0. Note that only three bacterial features were enriched in the high RFI group, while no preferential enrichment was detected in the low RFI group. **b** Relative abundance of three differentially enriched bacterial features. Results were plotted using box and whisker plots, in which the middle line denoted the median value and the lower and upper hinges represented the first and third quartiles, respectively. Whiskers extended from the hinge to the highest or lowest value no farther than 1.5× the interquartile range. Points outside of this range are considered outliers. Significance was calculated using Kruskal-Wallis test

LEfSe analysis was employed to identify differential enrichment of bacterial features between high and low RFI chickens using an LDA score of 2.0 as the threshold. In the ileum, two unidentified Peptostreptococcaceae F15 and F43 as well as S*ubdoligranulum* F6 were enriched in the high RFI group, although no bacteria were found to be enriched in low RFI chickens (Fig. 5a). Kruskal-Wallis test confirmed statistical significance (*P* < 0.05) with all three features in the ileum (Fig. 5b). It is noted that F15 and F43 are highly related differing by only one nucleotide along 403 nucleotides in the V3-V4 region of the 16S rRNA gene (data not shown). A BLAST search of the GenBank database revealed that F15 and F43 shared an approximately 98% identity to *Clostridium difficile*, *Intestinibacter bartlettii* (formally known as *Clostridium bartlettii*), and *Romboutsia ilealis*, all of which belong to cluster XI of *Clostridium* [35]. A BLAST search of *Subdoligranulum* F6 confirmed it 100% identical to *S. variabile*, a Ruminococcaceae family member (*Clostridium* cluster IV) initially reported in human feces [36].

**Fig. 6:**
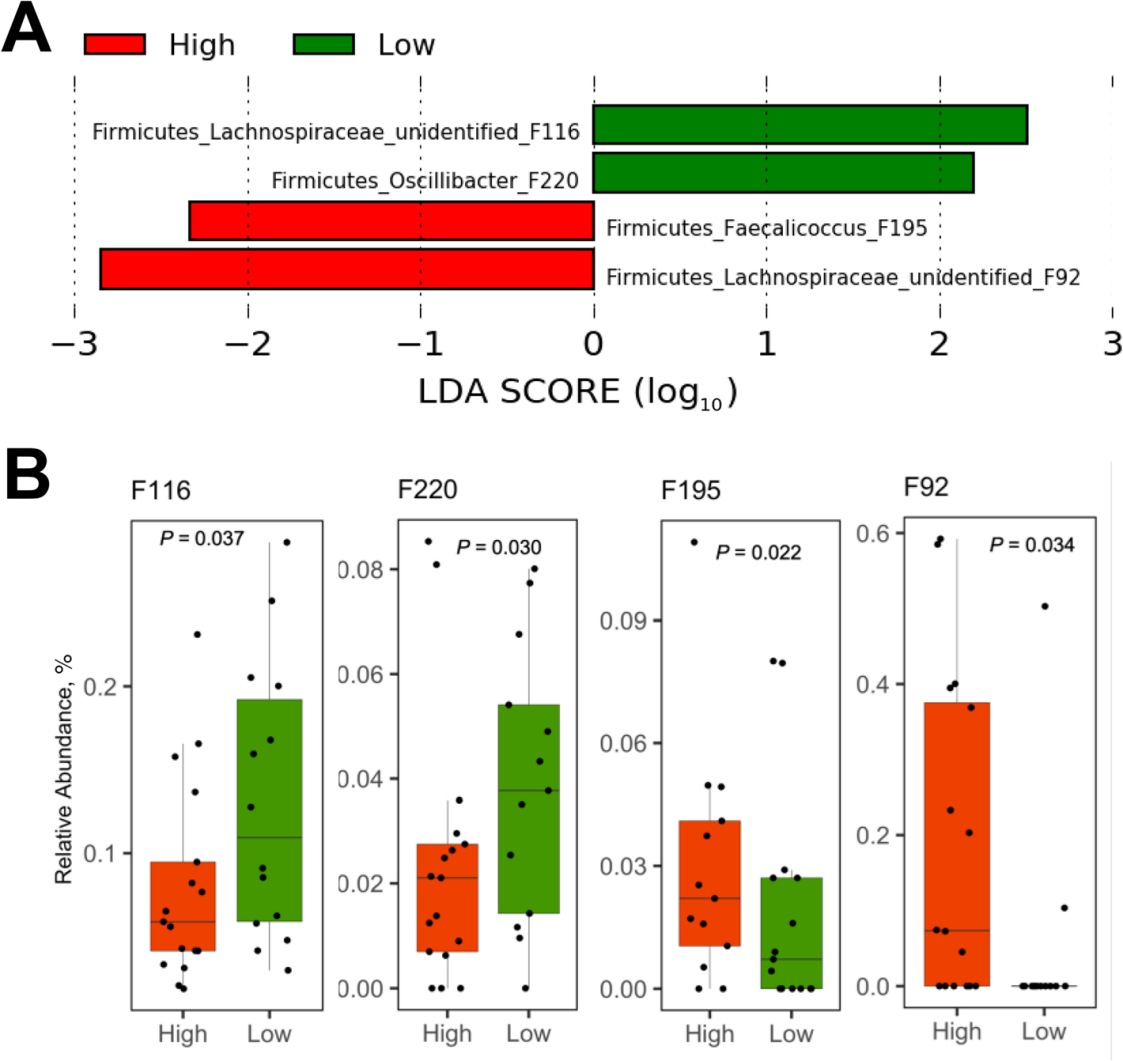
LEfSe analysis of the day-35 cecal microbiota of high and low RFI chickens. **a** Differential enrichment of the bacterial features was determined using LEfSe with a logarithmic LDA threshold of 2.0. **b** Relative abundance of five differentially enriched bacterial features. Results were plotted using box and whisker plots, in which the middle line denoted the median value and the lower and upper hinges represented the first and third quartiles, respectively. Whiskers extended from the hinge to the highest or lowest value no farther than 1.5 × the interquartile range. Points outside of this range are considered outliers. Significance was calculated using Kruskal-Wallis test

**Fig. 7:**
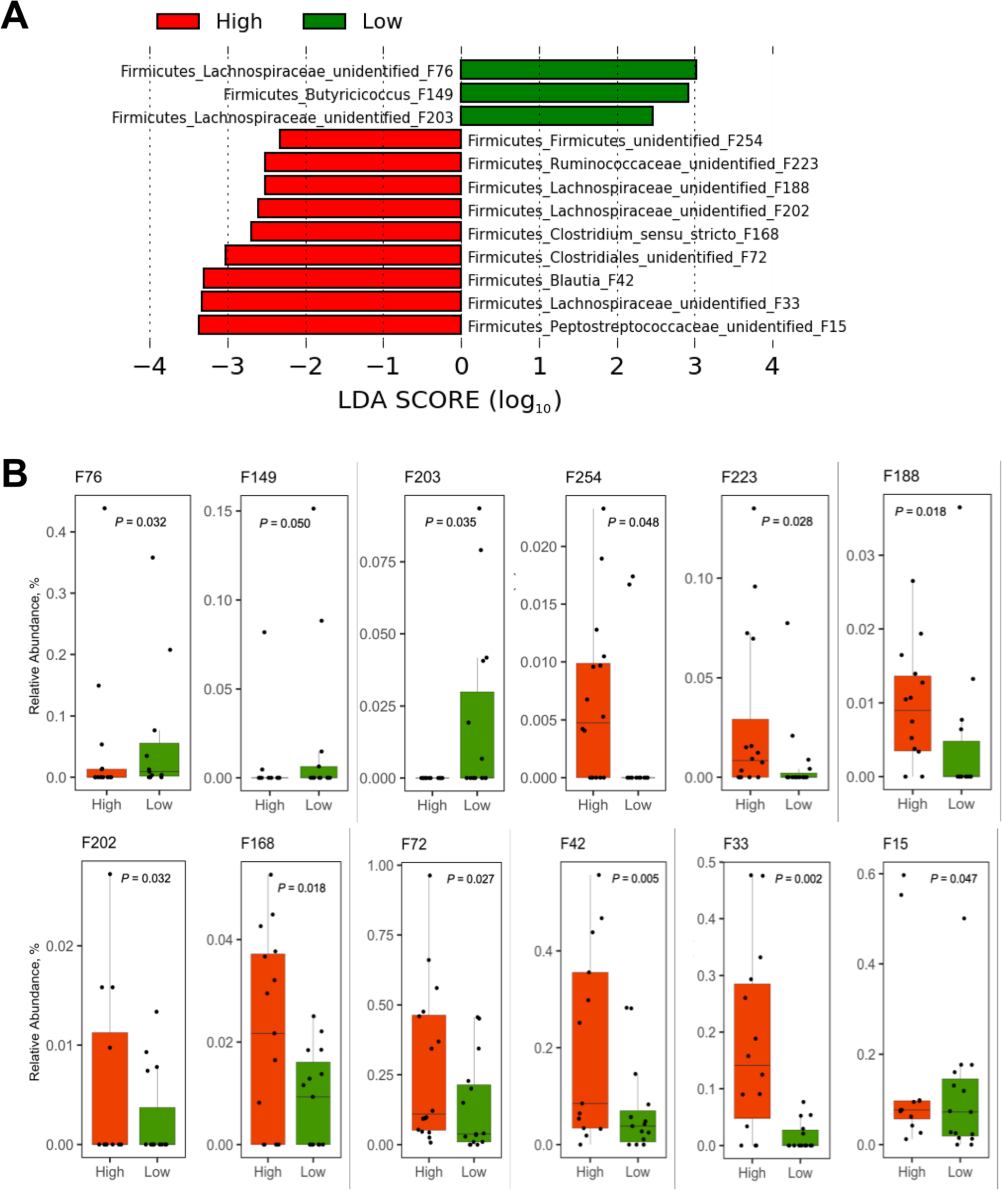
LEfSe analysis of the day-35 cloacal microbiota of high and low RFI chickens. **a** Differential enrichment of the bacterial features was determined using LEfSe with a logarithmic LDA threshold of 2.0. **b** Relative abundance of differentially enriched bacterial features. Results were plotted using box and whisker plots, in which the middle line denoted the median value and the lower and upper hinges represented the first and third quartiles, respectively. Whiskers extended from the hinge to the highest or lowest value no farther than 1.5 × the interquartile range. Points outside of this range are considered outliers. Significance was calculated using Kruskal-Wallis test

In the cecum, an unidentified Lachnospiraceae F116 (96.5% identical to *Blautia hominis* or *B. marasmi*) and *Oscillibacter* F220 (97.3% identical to *O. valericigenes*) were enriched in the low RFI group, while *Faecalicoccus* F195 (93.6% identical to *F. acidoformans*) and an unidentified Lachnospiraceae F92 were enriched in the high RFI group (Fig. 6a and b). In the cloaca, among a total of 12 differentially enriched features, an unidentified Lachnospiraceae member F76 and *Butyricicoccus* F149 (97.5% identical to *B. faecihominis*) were more abundant in the low RFI group, while an unidentified Peptostreptococcaceae F15, unidentified Lachnospiraceae F33, and *Blautia* F42 (98.3% identical to *B. Obeum*) were preferentially present in the high RFI group (Fig. 7a and b).

### Correlation between the intestinal microbiome and RFI

**Fig. 8:**
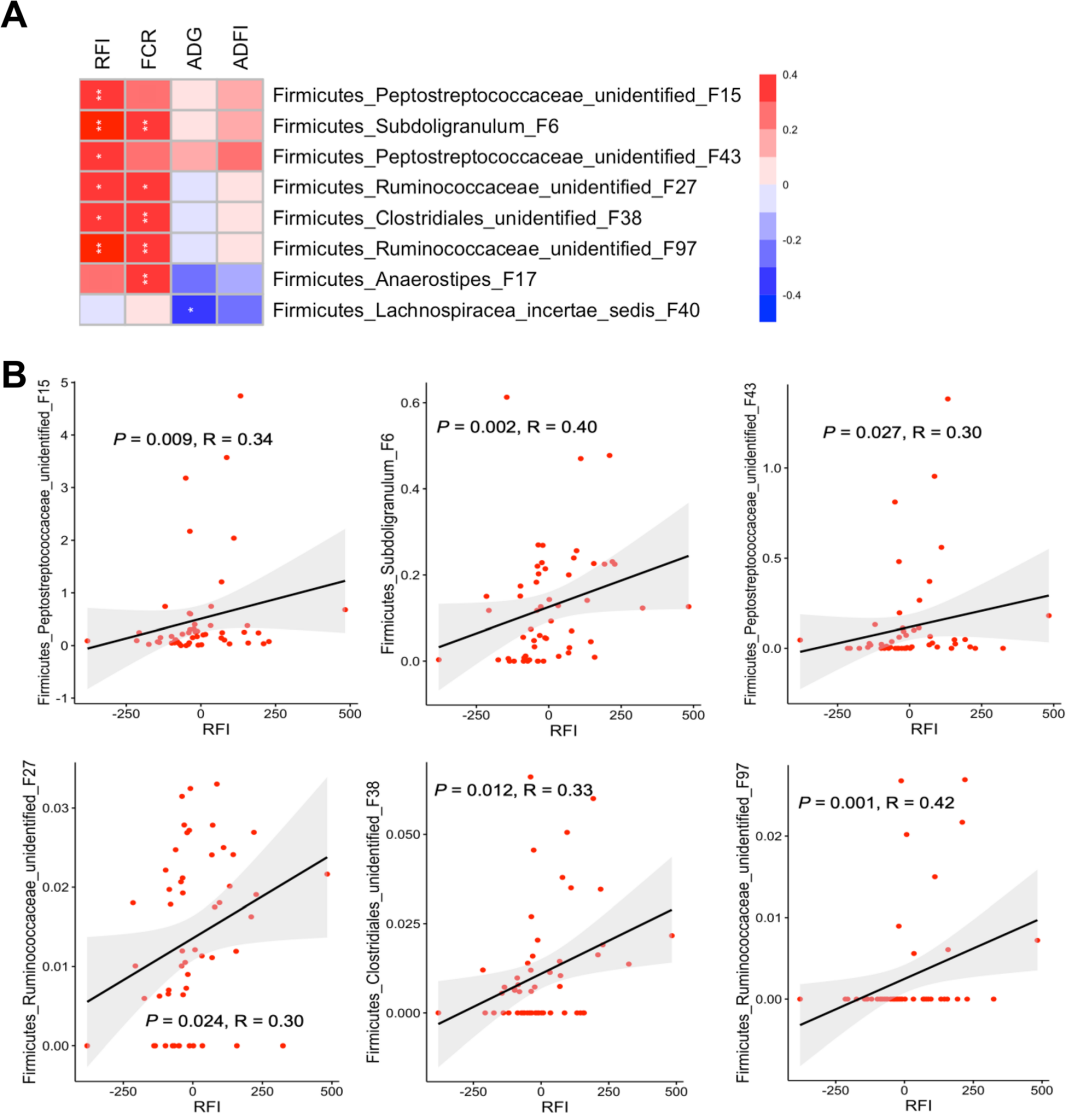
Spearman correlation between RFI and relative abundance of bacterial features in the ileum of day-35 chickens. All 56 ileal samples were used in Spearman’s rank correlation analysis and only those features with *P* < 0.05 and |R| ≥ 0.30 were shown (**a**). Note that there were no features showing a negative correlation with RFI. **b** Scatterplots of individual ileal bacterial features showing a significant correlation with RFI. P and R values were indicated for each feature. Solid line in the graph represented the line of best fit, while gray shading around the line indicated the 95% confidence interval. In a few cases, 1–3 extremely outlier samples were omitted for the sake of better visualization

**Fig. 9:**
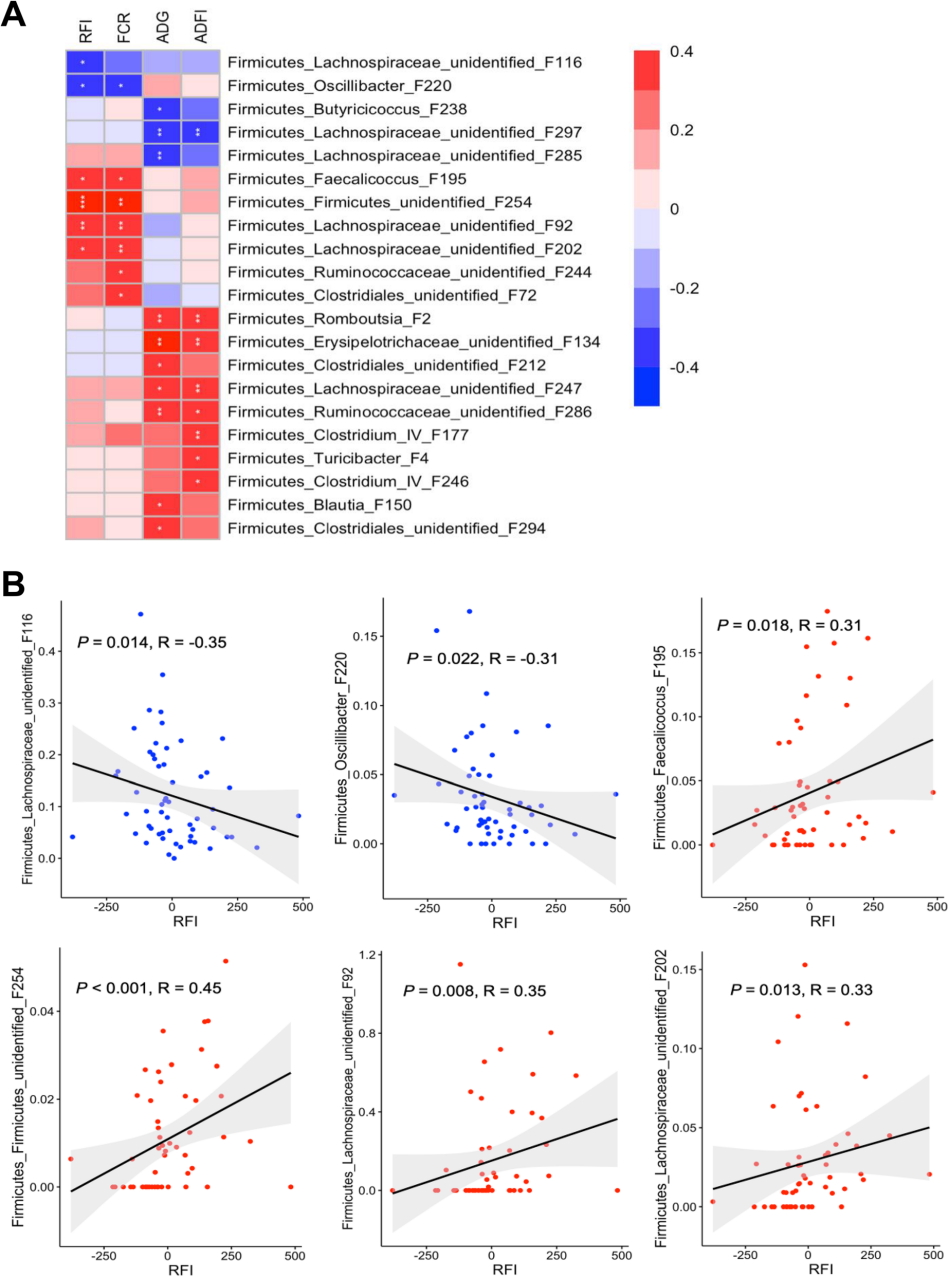
Spearman correlation between RFI and relative abundance of bacterial features in the cecum of day-35 chickens. All 56 cecal samples were used in Spearman’s rank correlation analysis and only those features with *P* < 0.05 and |R| ≥ 0.30 were shown (**a**). **b** Scatterplots of individual ileal bacterial features showing a significant correlation with RFI. P and R values were indicated for each feature. Solid line in the graph represented the line of best fit, while gray shading around the line indicated the 95% confidence interval

**Fig. 10:**
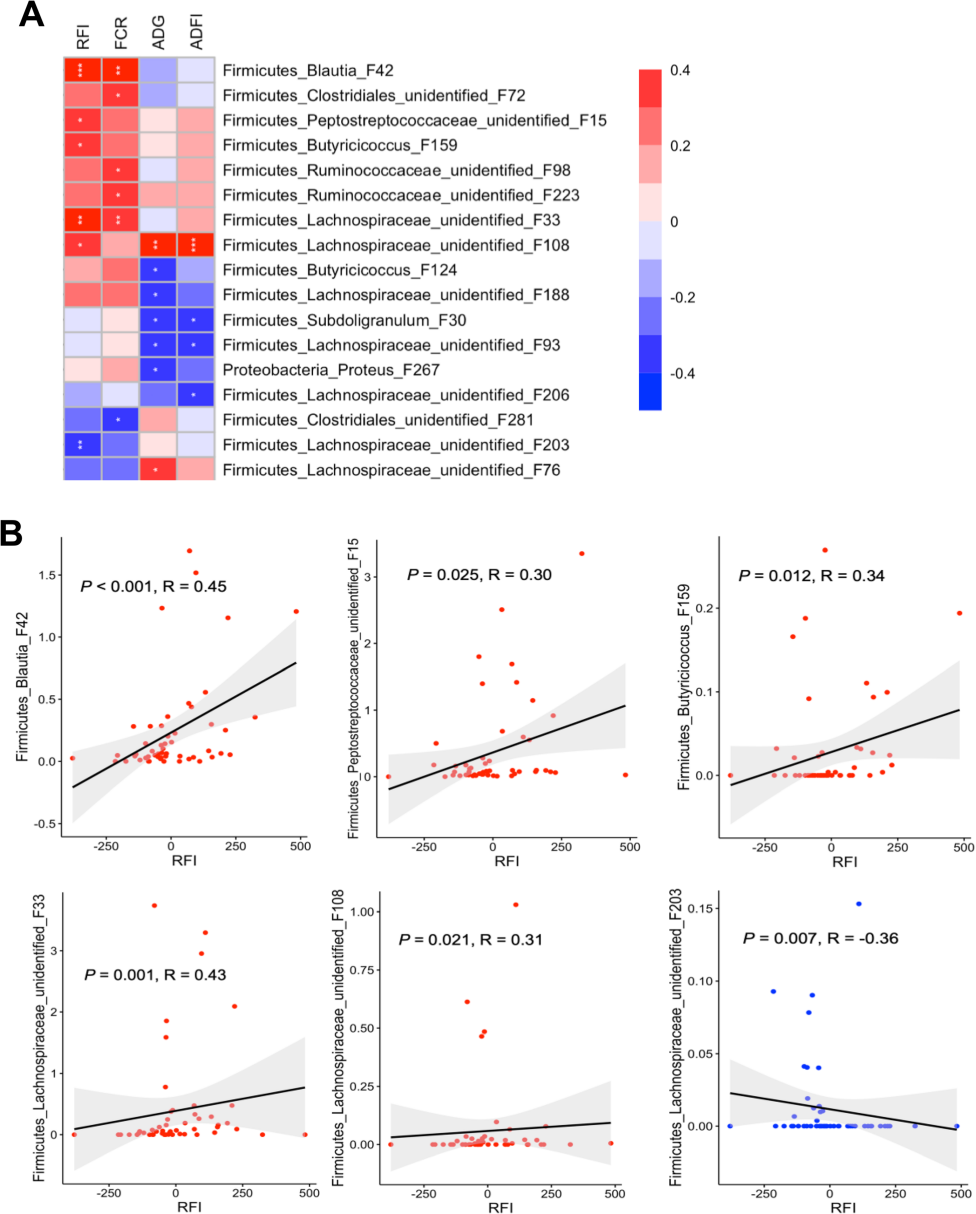
Spearman correlation between RFI and relative abundance of bacterial features in the cloaca of day-35 chickens. **a** All 56 cloacal samples were used in Spearman’s rank correlation analysis and only those features with *P* < 0.05 and |R| ≥ 0.30 were shown. **b** Scatterplots of individual ileal bacterial features showing a significant correlation with RFI. P and R values were indicated for each feature. Solid line in the graph represented the line of best fit, while gray shading around the line indicated the 95% confidence interval. In a few cases, 1–3 extremely outlier samples were omitted for the sake of better visualization

To further reveal the correlations between RFI and relative abundances of all bacterial features in the ileal, cecal, and cloacal samples of all 54 chickens, Spearman correlation analysis was performed. A total of 6 features showed significant positive correlations (*P* < 0.05) with RFI in the ileum (Fig. 8a), with R values ranging from 0.3 to 0.42 (Fig. 8b), albeit with no bacteria showing a negative correlation. In the cecum, two features were significantly negatively correlated with RFI (*P* < 0.05), with another four showing a significant positive correlation (*P* < 0.05) (Fig. 9a). The |R| values of these features ranged from 0.31 to 0.45 (Fig. 9b). In the cloaca, a total of six features were found to be significantly associated with RFI (*P* < 0.05), with one displaying a negative correlation and five showing a positive correlation (Fig. 10a). Among the most strongly associated features were an unidentified Peptostreptococcaceae F15 (*P* = 0.009, *R* = 0.34) and *S. variabile* F6 (*P* = 0.002, *R* = 0.40) in the ileum (Fig. 8b); an unidentified Firmicutes F254 (*P* < 0.001, *R* = 0.45) and an unidentified Lachnospiraceae F92 (*P* = 0.008, *R* = 0.35) in the cecum (Fig. 9b); and *Blautia* F42 (*P* < 0.001, *R* = 0.45) and unidentified Lachnospiraceae F33 (*P* = 0.001, *R* = 0.43) and F203 (*P* = 0.007, *R* = − 0.36) in the cloaca (Fig. 10b). It is noteworthy that Lachnospiraceae F33, F76, and F92 are closely related (Fig. S1) and a BLAST search revealed that all three showed 96–97% identity to *Mediterraneibacter faecis*, *M. lactaris*, or *M. torques* [37].

Because of the availability of individual FCR, ADG, and ADFI values, we also performed Spearman correlation analysis between each phenotype and the intestinal microbiota profiles. Most of the bacterial features that were correlated with RFI were also similarly correlated with FCR, in the ileum (Fig. 8a), cecum (Fig. 9a), and cloaca (Fig. 10a), which is perhaps not surprising, given that the two groups of birds selected in this study were similarly segregated not only in RFI (*P* < 0.001), but also in FCR (*P* < 0.001) (compare Fig. 1b to f). However, a largely different group of bacteria were strongly correlated with ADG and ADFI in each of the three intestinal locations (Figs. 8a, 9a, and 10a).

## Discussion

Identifying RFI-related intestinal microbes is critically important in understanding the mechanisms involved in feed efficiency. It also provides opportunities to manipulate the intestinal microbiota to enhance the profitability and sustainability of the livestock production. A possible association between the intestinal microbiota and RFI in broiler chickens has been explored. However, the microbiota differences at the genus or OTU level are generally subtle between high and low RFI chickens, and it is perhaps not surprising the outcomes are inconsistent among different studies [19–23]. Therefore, there is a need to address the discrepancies and further examine whether RFI-associated bacteria could be more reliably revealed at the subspecies level. In this study, we analyzed the ileal, cecal, and cloacal microbiotas, simultaneously, for their relationships with feed efficiency of broilers and explored for the first time QIIME 2’s Deblur method of denoising allowing for single-nucleotide resolution in differentiating bacterial features. As a result, we have identified a number of features in the ileum, cecum, and cloaca that are strongly linked to RFI. All seven bacterial features that are differentially enriched in the ileal and cecal microbiota of high or low RFI chickens based on LEfSe analysis are significantly correlated with RFI. Three differentially enriched cloacal bacterial features also show a significant correlation with RFI.

Most of the bacteria that are strongly associated in either high or low RFI chickens belong to Clostridiales, which are is a highly diverse order of obligate anaerobes that ferment host-indigestible plant polysaccharides into SCFAs [38, 39]. Clostridia are abundant in soil and also in the gastrointestinal (GI) tract, representing up to 20% of uncultured genomes in the human GI tract [40]. Among families of Clostridiales are Clostridiaceae (*Clostridium* cluster I), Ruminococcaceae (cluster IV), Oscillospiraceae (cluster IV), Peptostreptococcaceae (cluster XI), and Lachnospiraceae (cluster XIVa) [41]. Lachnospiraceae and Ruminococcaceae are highly abundant in poultry and are particularly effective at degrading cellulose and other host-indigestible polysaccharides [42]. Consistently, we found that *Oscillibacter* F220 (family Oscillospiraceae) in the cecum and *Butyricicoccus* F149 (family Clostridiaceae) in the cloaca are enriched in low RFI chickens and positively associated with feed efficiency. It will be important to experimentally verify the differences in the digestibility of the nutrients such as carbohydrates, proteins, and/or lipids between high and low RFI chickens. Metagenomics or metatranscriptomics studies will also be needed to confirm an enrichment of the genes involved in digestion and utilization of various nutrients in low RFI animals.

However, to our surprise, a majority of other SCFA-producing clostridia are more abundant in high RFI chickens and negatively correlated with feed efficiency. For example, two unidentified and closely-related Peptostreptococcaceae F15 and F43 show a differential enrichment in high RFI and significant positive correlation with RFI in the ileum, meaning that both are negatively associated with feed efficiency. F15 is also negatively correlated with feed efficiency in the cloaca as well. F15 and F43 are highly related to *Clostridium* cluster XI bacterial such as *C. difficile*, *I. bartlettii*, and *R. ilealis*. Although *C. difficile* is a well-known enteric pathogen, little is known about *I. bartlettii* or *R. ilealis*. Analysis of the *R. ilealis* genome revealed its limited capacity to synthesize amino acids and vitamins but with the ability to utilize relatively simple carbohydrates such as glucose, L-fucose and fructo-oligosaccharides [35]. The reason why Peptostreptococcaceae F15 and F43 reduce feed efficiency remains to be investigated, although *I. bartlettii* appears to be more abundant in the ileum of turkeys with heavier BW [43].

*S. variabile* F6 (family Ruminococcaceae) is enriched in the ileum of high-RFI, low-feed efficiency chickens with a significant negative correlation with feed efficiency. It is perhaps not surprising because *S. variabile* is known to be differentially enriched in the children with food sensitization [44] and correlated positively with lipid metabolic dysfunction and inflammatory response in the ileum of pigs [45]. Several other clostridial bacteria including two unidentified members of Ruminococcaceae F27 and F97 in the ileum, *Faecalicoccus* F195 (family Erysipelotrichaceae) in the cecum, and an unidentified Clostridiales F72 in the cloaca also show a significant positive correlation with RFI. The reason for a negative correlation between these SCFA-producing bacteria and feed efficiency remains to be further investigated. These bacteria may influence feed efficiency beyond mere fermentation of plant polysaccharides. Alternatively, they may not directly impact feed efficiency, but changes in the microbiota of high RFI chickens may lead to their overgrowth.

Our results have also clearly revealed that multiple closely-related members of the Lachnospiraceae family are significant associated with RFI. Among those differentially enriched Lachnospiraceae that also show a strong correlation with RFI, to our surprise several features (e.g., F76, F116, and F203) are negatively correlated with RFI, while others (e.g., F33, F42, and F92) show a positive correlation. Alignment of these six sequences reveals a minimum 91.3% identity (Fig. S1). In particular, F33, F76, and F92 are highly related, showing 96.5–97.8% identities among each other*.* Although F33, F76, and F92 could otherwise be grouped as a single OTU, their impact on feed efficiency is totally opposite. While F33 in the cloaca and F92 in the cecum are negatively correlated with feed efficiency, F76 is highly enriched in the cloaca of low RFI chickens with a strong tendency to be positively correlated with feed efficiency.

Similarly, two related *Blautia* F42 and F116 of the Lachnospiraceae family also show an opposite association with feed efficiency. Albeit with 94.8% identity with each other (Fig. S1), F42 is negatively correlated with feed efficiency in the cloaca, while F116 has an opposite association in the cecum. These results clearly demonstrate the advantage of differentiating the intestinal microbes to the single-nucleotide resolution at the species, subspecies, or strain level. Otherwise, the physiological effect of some of the bacteria could be masked. In fact, our findings are consistent with the well-known fact that functional variations exist among different species of a bacterial family or even different strains of the same bacterial species. However, because of limitations of the current nonredundant (NR) and 16S rRNA gene database in GenBank, most of the newly identified RFI-associated bacterial features are found to be 100% identical to uncultured and unclassified 16S rRNA sequences. Only *Subdoligranulum* F6 can be unequivocally assigned to *S. variabile*, and we failed to assign a specific bacterial species or strain name to all other features.

Notably, the RFI-associated bacteria revealed in this study are mostly different from several earlier attempts [19–23]. In fact, earlier results vary from study to study as well. For example, among three different earlier studies, *Dorea* (family Lachnospiraceae) [19], an *Anaerobacterium* OTU (family Ruminococcaceae) [21], two *Lactobacillus* OTUs [21], and the Christensenellaceae family [23] have been found to be significantly associated with low RFI chickens, while two *Gracilibacter* OTUs and a *Clostridium* OTU are associated with high RFI chickens [21]. The results are even different between two animal experiments being duplicated at two different locations [19].

These apparently inconsistent outcomes may potentially be due to dietary and environmental differences among different flocks used in the studies. Bacteria present on eggshell and in the early environment such as feed and bedding are known to contribute significantly to the intestinal microbiota [46, 47]. Secondly, the discrepancies among different studies may be due to relatively low stringency of selection for high and low RFI birds. In our study, 32 out of 56 chickens or 57% chickens were selected to form high and low RFI groups, whereas approximately 50% chickens were chosen in most earlier studies [20–23] and 20% chickens were selected in a fifth study [19]. It is possible that a higher stringency of selection for extreme high and low RFI chickens may lead to more reproducible results. Thirdly, the discrepancies among the studies may occur because it is the function, but not the composition, of the intestinal microbiota that dictates feed efficiency. Although different studies have revealed an association between different bacteria with RFI, it is plausible that consistent functional alternations might occur between high and low RFI animals. Techniques such as metagenomics, metabolomics, metatranscriptomics, and metaproteomics [48, 49] will be useful to reveal the differences in the functional capacity of the intestinal microbiota between high- and low-performing animals.

## Conclusion

The intestinal microbiota is a complex community of microorganisms and variations in the structure and function of individual animals have a profound impact on the health and performance outcomes of host animals. In this study, we have identified a number of bacteria that are strongly associated with feed efficiency in three different GI locations of broiler chickens. Among those newly-identified RFI-associated bacteria, most belong to the order of Clostridiales. Importantly, we revealed the complexity of the intestinal microbiota that are linked to RFI. While a few Lachnospiraceae family members are positively correlated with feed efficiency, other closely related bacteria have an opposite impact, highlighting a need to differentiate the bacteria to the species, subspecies, and even stain levels. Apparently, this work enhances our understanding of the link between the intestinal microbiome and feed efficiency in broilers. Identification of performance-associated bacterial taxa marks a first step towards developing efficacious, cost-effective prebiotic or probiotic formulations to replace antibiotics as feed additives for growth promotion and disease prevention. It will be beneficial to further increase the selection pressure for high- and low-performing animals with a larger difference in RFI values in future studies. It is also important to investigate not only the composition, but also the functional potential of the intestinal microbiota and evaluate their functional correlations with feed efficiency in the future.

## Supplementary Information

**Additional file 1: Table S1.** Composition of the experimental diets. **Table S2.** Relative abundance (%) of the intestinal bacterial genera in day-35 high and low RFI chickens. **Table S3.** Relative abundance (%) of the intestinal bacterial features in day-35 high and low RFI chickens. **Figure S1.** Multiple sequence alignment (A) and percent identity matrix (B) among six closely related Lachnospiraceae family members that are strongly associated with residual feed intake.

## Data Availability

All data generated or analyzed during this study are included in this published article and its supplementary information files.

## Abbreviations

ADFI: Average daily feed intake
ADG: Average daily gain
ASV: Amplicon sequence variant
BW: Body weight
CSS: Cumulative sum scaling
FCR: Feed conversion ratio
GI: Gastrointestinal
LEfSe: Linear discriminant analysis effect size
MMW: Mid-test metabolic weight
OTU: Operational taxonomic unit
PERMANOVA: Permutational multivariate analysis of variance
RFI: Residual feed intake
SCFA: Short-chain fatty acid
TBWG: Total body weight gain
TFI: Total feed intake
