# Supplementary material for: Linkage between the intestinal microbiota and residual feed intake in broiler chickens
Source: J Anim Sci Biotechnol. 2021 Feb 11;12:22. doi: 10.1186/s40104-020-00542-2 (PMC7879522; doi:10.1186/s40104-020-00542-2)
Supplement: Supplementary file 1 — Additional file 1: Table S1. Composition of the experimental diets. Table S2. Relative abundance (%) of the intestinal bacterial genera in day-35 high and low RFI chickens. Table S3. Relative abundance (%) of the intestinal bacterial features in day-35 high and low RFI chickens. Figure S1. Multiple sequence alignment (A) and percent identity matrix (B) among six closely related Lachnospiraceae family members that are strongly associated with residual feed intake. [file 40104_2020_542_MOESM1_ESM.docx]

**Supplementary information**

**Linkage between the intestinal microbiota and residual feed intake in broiler chickens**

Jing Liu^1,†^, Sydney N. Stewart^1,†^, Kelsy Robinson^1,‡^, Qing Yang^1^, Wentao Lyu^1,2^, Melanie A. Whitmore^1^, and Guolong Zhang^1,#^

^1^ Department of Animal and Food Sciences, Oklahoma State University, Stillwater, Oklahoma, USA

^2^ Institute of Quality and Standards for Agro-products, Zhejiang Academy of Agricultural Sciences, Hangzhou, China

^†^ J.L. and S.N.S contributed equally to this article

^‡^ Current address: Poultry Production and Product Safety Research Unit, USDA-Agricultural Research Service (ARS), Fayetteville, Arkansas, USA.

^#^ Correspondence: Guolong Zhang, zguolon@okstate.edu

**Table S1. Composition of the experimental diets**

| Ingredients^1^ | Starter (D0 – D8) | Grower (D9 – D18) | Finisher (D19 – D35) |
| --- | --- | --- | --- |
| Yellow corn, % | 52.8 | 57.1 | 60.4 |
| Soybean meal, % | 39.7 | 34.9 | 30.7 |
| Soybean oil, % | 3.5 | 4.2 | 5.2 |
| Dicalcium phosphate (18.5%), % | 2.04 | 1.81 | 1.68 |
| Limestone, % | 1.06 | 0.97 | 0.95 |
| Salt, % | 0.48 | 0.48 | 0.43 |
| *DL*-Methionine, % | 0.16 | 0.22 | 0.20 |
| Threonine, % | 0.05 | 0.07 | 0.11 |
| Poultry premix, NB 3000^1^, % | 0.03 | 0.25 | 0.25 |
| Total ME, kcal/kg | 2987.60 | 3082.20 | 3176.80 |
| Total CP, % | 21.50 | 19.61 | 18.00 |

^1^ Supplied per kilogram of diet: manganese, 0.02%; zinc, 0.02%; iron, 0.01%; copper, 0.0025%; iodine, 0.0003%; selenium, 0.00003%; folic acid, 0.69 mg; choline, 386 mg; riboflavin, 6.61 mg; biotin, 0.03 mg; vitamin B_6_, 1.38 mg; niacin, 27.56 mg; pantothenic acid, 6.61 mg; thiamine, 2.20 mg; manadione, 0.83 mg; vitamin B_12_, 0.01 mg; vitamin E, 16.53 IU; vitamin D_3_, 2,133 ICU; vitamin A, 7,716 IU.

**Table S2**. **Relative abundance (%) of the intestinal bacterial genera in day-35 high and low RFI chickens**

| Genera | High RFI | Low RFI | *P*-value | FDR |
| --- | --- | --- | --- | --- |
| Ileum | | | | |
| *Lactobacillus* | 35.69 | 32.03 | 0.355 | 0.568 |
| *Romboutsia* | 21.06 | 29.68 | 0.417 | 0.568 |
| *Enterococcus* | 18.72 | 19.39 | 0.396 | 0.568 |
| *Turicibacter* | 12.98 | 8.22 | 0.748 | 0.748 |
| *Streptococcus* | 0.49 | 7.16 | 0.299 | 0.560 |
| *Peptostreptococcaceae*_unidentified | 6.14 | 0.16 | 0.013 * | 0.112 |
| *Escherichia/Shigella* | 3.03 | 2.18 | 0.584 | 0.626 |
| *Lachnospiraceae*_unidentified | 0.45 | 0.36 | 0.117 | 0.351 |
| *Subdoligranulum* | 0.26 | 0.14 | 0.020 * | 0.112 |
| *Terrisporobacter* | 0.26 | 0.05 | 0.199 | 0.427 |
| *Faecalibacterium* | 0.18 | 0.14 | 0.157 | 0.392 |
| *Ruminococcaceae*_unidentified | 0.17 | 0.10 | 0.030 * | 0.112 |
| *Anaerostipes* | 0.06 | 0.04 | 0.027 * | 0.112 |
| *Bacteroides* | 0.06 | 0.05 | 0.558 | 0.626 |
| *Streptophyta* | 0.03 | 0.07 | 0.466 | 0.583 |
| Cecum | | | | |
| *Lachnospiraceae*_unidentified | 22.18 | 23.15 | 0.462 | 0.865 |
| *Lactobacillus* | 14.26 | 11.77 | 0.664 | 0.897 |
| *Faecalibacterium* | 13.23 | 10.68 | 0.355 | 0.865 |
| *Ruminococcaceae*_unidentified | 10.54 | 10.67 | 0.985 | 0.985 |
| *Subdoligranulum* | 8.12 | 12.84 | 0.193 | 0.722 |
| *Bacteroides* | 4.67 | 5.34 | 0.748 | 0.897 |
| *Clostridiales*_unidentified | 3.64 | 2.93 | 0.193 | 0.722 |
| *Romboutsia* | 3.14 | 3.40 | 0.777 | 0.897 |
| *Enterococccus* | 2.44 | 3.54 | 0.462 | 0.865 |
| *Blautia* | 2.67 | 3.53 | 0.720 | 0.897 |
| *Anaerostipes* | 1.88 | 1.95 | 0.955 | 0.985 |
| *Butyricicoccus* | 1.82 | 1.55 | 0.375 | 0.865 |
| *Lachnospiraceae*_incertae_sedis | 1.42 | 1.48 | 0.610 | 0.897 |
| Ruminococcus2 | 1.65 | 1.13 | 0.168 | 0.722 |
| *Clostridium*_IV | 1.08 | 0.90 | 0.168 | 0.722 |
| Cloaca |  |  |  |  |
| *Lactobacillus* | 17.05 | 17.62 | 0.895 | 0.895 |
| Lachnospiraceae_unidentified | 14.40 | 14.81 | 0.895 | 0.895 |
| *Romboutsia* | 13.50 | 11.63 | 0.865 | 0.895 |
| *Enterococcus* | 7.72 | 13.16 | 0.584 | 0.895 |
| *Subdoligranulum* | 5.12 | 7.39 | 0.692 | 0.895 |
| *Bacteroides* | 7.42 | 4.67 | 0.146 | 0.895 |
| *Turicibacter* | 9.23 | 2.57 | 0.317 | 0.895 |
| *Faecalibacterium* | 6.10 | 4.55 | 0.299 | 0.895 |
| Ruminococcaceae_unidentified | 4.81 | 4.60 | 0.806 | 0.895 |
| *Streptococcus* | 0.56 | 6.36 | 0.533 | 0.895 |
| Clostridiales_unidentified | 2.78 | 2.31 | 0.417 | 0.895 |
| *Anaerostipes* | 1.50 | 1.93 | 0.865 | 0.895 |
| *Escherichia/Shigella* | 1.26 | 1.82 | 0.835 | 0.895 |
| *Blautia* | 1.46 | 1.19 | 0.417 | 0.895 |
| *Lachnospiraceae*_incertae_sedis | 0.85 | 0.81 | 0.835 | 0.895 |

**Note**: Mean relative abundance (%) of the top 15 genera are shown, with 15 high and 17 low RFI samples per intestinal segment. Statistical significance was determined using the Kruskal-Wallis test with Benjamini-Hochberg correction. False discovery rate (FDR) is shown.

**Table S3. Relative abundance (%) of the intestinal bacterial features in day-35 high and low RFI chickens**

| Features | High RFI | Low RFI | *P*-value | FDR |
| --- | --- | --- | --- | --- |
| Ileum | | | | |
| Firmicutes_Lactobacillus_F1 | 29.18 | 27.26 | 0.462 | 0.674 |
| Firmicutes_Romboutsia_F2 | 21.05 | 29.68 | 0.417 | 0.674 |
| Firmicutes_Enterococcus_F3 | 18.70 | 19.38 | 0.396 | 0.674 |
| Firmicutes_Turicibacter_F4 | 12.97 | 8.21 | 0.748 | 0.876 |
| Firmicutes_Streptococcus_F8 | 0.49 | 7.16 | 0.335 | 0.674 |
| Firmicutes_Lactobacillus_F10 | 3.19 | 2.89 | 0.865 | 0.927 |
| Firmicutes_Peptostreptococcaceae_unidentified_F15 | 5.16 | 0.14 | 0.018 * | 0.137 |
| Proteobacteria_Escherichia/Shigella_F12 | 3.03 | 2.18 | 0.584 | 0.730 |
| Firmicutes_Lactobacillus_F24 | 1.13 | 0.92 | 0.788 | 0.876 |
| Firmicutes_Lactobacillus_F26 | 1.25 | 0.25 | 0.433 | 0.674 |
| Firmicutes_Lactobacillus_F22 | 0.73 | 0.68 | 0.472 | 0.674 |
| Firmicutes_Peptostreptococcaceae_unidentified_F43 | 0.98 | 0.02 | 0.009 * | 0.137 |
| Firmicutes_Terrisporobacter_F75 | 0.26 | 0.05 | 0.199 | 0.597 |
| Firmicutes_Subdoligranulum_F6 | 0.18 | 0.09 | 0.003 * | 0.115 |
| Firmicutes_Lactobacillus_F102 | 0.16 | 0.02 | 0.252 | 0.674 |
| Firmicutes_Ruminococcaecea_unidentified_F11 | 0.09 | 0.06 | 0.155 | 0.597 |
| Firmicutes_Faecalibacterium_F9 | 0.07 | 0.07 | 0.985 | 0.985 |
| Firmicutes_Faecalibacterium_F5 | 0.06 | 0.04 | 0.570 | 0.730 |
| Firmicutes_Lachnospiraceae_unidentified_F14 | 0.05 | 0.04 | 0.375 | 0.674 |
| Firmicutes_Lachnospiraceae_unidentified_F16 | 0.05 | 0.03 | 0.060 | 0.362 |
| Firmicutes_Anaerostipes_F17 | 0.03 | 0.03 | 0.015 * | 0.137 |
| Firmicutes_Lachnospiraceae_unidentified_F19 | 0.03 | 0.06 | 0.772 | 0.876 |
| Firmicutes_Lachnospiraceae_unidentified_F20 | 0.05 | 0.03 | 0.121 | 0.597 |
| Cyanobacteria_Chloroplast_Streptophyta_F192 | 0.02 | 0.05 | 0.466 | 0.674 |
| Firmicutes_Lachnospiraceae_unidentified_F18 | 0.04 | 0.03 | 0.416 | 0.674 |
| Firmicutes_Faecalibacterium_F21 | 0.05 | 0.02 | 0.174 | 0.597 |
| Firmicutes_Bacteroides_F7 | 0.03 | 0.02 | 0.450 | 0.674 |
| Firmicutes_Subdoligranulum_F30 | 0.03 | 0.02 | 0.195 | 0.597 |
| Firmicutes_Lachnospiraceae_unidentified_F23 | 0.03 | 0.03 | 0.544 | 0.730 |
| Firmicutes_Clostridium_sensus_stricto_F227 | 0.04 | 0.00 | 0.960 | 0.985 |
| Cecum | | | | |
| Firmicutes_Lactobacillus_F1 | 11.78 | 10.28 | 0.985 | 0.985 |
| Firmicutes_Subdoligranulum_F6 | 4.68 | 8.89 | 0.439 | 0.894 |
| Firmicutes_Faecalibacterium_F5 | 6.76 | 5.45 | 0.748 | 0.894 |
| Firmicutes_Faecalibacterium_F9 | 5.10 | 4.25 | 0.637 | 0.894 |
| Firmicutes_Ruminococcaceae_unidentied_F11 | 4.27 | 4.60 | 0.610 | 0.894 |
| Firmicutes_Romboutsia_F2 | 3.14 | 3.40 | 0.777 | 0.894 |
| Firmicutes_Enterococcus_F3 | 2.43 | 3.52 | 0.462 | 0.894 |
| Bacteroidetes_Bacteroides_F7 | 2.42 | 2.81 | 0.748 | 0.894 |
| Firmicutes_Lachnospiraceae_unidentified_F20 | 1.40 | 2.59 | 0.558 | 0.894 |
| Firmicutes_Lachnospiraceae_unidentified_F18 | 1.85 | 1.98 | 0.439 | 0.894 |
| Bacteroidetes_Bacteroides_F13 | 1.68 | 1.94 | 0.664 | 0.894 |
| Firmicutes_Lachnospiraceae_unidentified_F16 | 2.30 | 1.18 | 0.558 | 0.894 |
| Firmicutes_Lachnospiraceae_unidentified_F19 | 0.60 | 2.96 | 0.116 | 0.894 |
| Firmicutes_Lachnospiraceae_unidentified_F14 | 1.76 | 1.64 | 0.439 | 0.894 |
| Firmicutes_Subdoligranulum_F25 | 1.66 | 1.37 | 0.834 | 0.894 |
| Firmicutes_Anaerostipes_F17 | 1.46 | 1.57 | 0.777 | 0.894 |
| Firmicutes_Lachnospiraceae_unidentified_F23 | 1.49 | 1.30 | 0.610 | 0.894 |
| Firmicutes_Ruminococcaceae_unidentified_F27 | 1.14 | 1.35 | 0.664 | 0.894 |
| Firmicutes_Subdoligranulum_F31 | 1.24 | 0.91 | 0.954 | 0.985 |
| Firmicutes_Subdoligranulum_F30 | 0.54 | 1.67 | 0.739 | 0.894 |
| Firmicutes_Faecalibacterium_F21 | 1.12 | 0.82 | 0.625 | 0.894 |
| Firmicutes_Turicibacter_F4 | 1.18 | 0.43 | 0.533 | 0.894 |
| Firmicutes_Ruminococcaceae_unidentified_F34 | 0.91 | 0.74 | 0.485 | 0.894 |
| Firmicutes_Lachnospiraceae_unidentified_F33 | 0.89 | 0.70 | 0.146 | 0.894 |
| Firmicutes_Lachnospiraceae_incertae_sedis_F32 | 0.93 | 0.58 | 0.213 | 0.894 |
| Firmicutes_Lachnospiraceae_unidentified_F35 | 0.83 | 0.68 | 0.157 | 0.894 |
| Firmicutes_Ruminococcus2_F28 | 0.92 | 0.58 | 0.156 | 0.894 |
| Firmicutes_Butyricoccus_F36 | 0.77 | 0.71 | 0.610 | 0.894 |
| Firmicutes_Ruminococcaceae_unidentified_F37 | 0.65 | 0.82 | 0.806 | 0.894 |
| Firmicutes_Lachnospiraceae_incertae_sedis_F40 | 0.48 | 0.88 | 0.089 | 0.894 |
| Cloaca |  |  |  |  |
| Firmicutes_Lactobacillus_F1 | 14.17 | 15.05 | 0.835 | 0.985 |
| Firmicutes_Romboutsia_F2 | 13.50 | 11.62 | 0.865 | 0.985 |
| Firmicutes_Enterococcus_F3 | 7.71 | 13.16 | 0.584 | 0.985 |
| Firmicutes_Turicibacter_F4 | 9.23 | 2.57 | 0.317 | 0.985 |
| Firmicutes_Subdoligranulum_F6 | 3.16 | 5.40 | 0.835 | 0.985 |
| Firmicutes_Streptococcus_F8 | 0.56 | 6.36 | 0.509 | 0.985 |
| Bacteroidetes_Bacteroides_F7 | 3.76 | 2.48 | 0.146 | 0.985 |
| Firmicutes_Faecalibacterium_F5 | 3.44 | 2.48 | 0.193 | 0.985 |
| Bacteroidetes_Bacteroides_F13 | 2.72 | 1.65 | 0.109 | 0.985 |
| Firmicutes_Ruminococcaceae_unidentified_F11 | 2.18 | 1.99 | 0.865 | 0.985 |
| Firmicutes_Faecalibacterium_F9 | 2.24 | 1.76 | 0.290 | 0.985 |
| Firmicutes_Lachnospiraceae_unidentified_F19 | 0.59 | 3.00 | 0.290 | 0.985 |
| Proteobacteria_Escherichia/Shigella_F12 | 1.26 | 1.82 | 0.835 | 0.985 |
| Firmicutes_Anaerostipes_F17 | 1.25 | 1.68 | 0.835 | 0.985 |
| Firmicutes_Lachnospiraceae_unidentified_F20 | 0.85 | 1.91 | 0.720 | 0.985 |
| Firmicutes_Lachnospiraceae_unidentified_F14 | 1.39 | 1.29 | 0.336 | 0.985 |
| Firmicutes_Lactobacillus_F10 | 1.19 | 1.40 | 0.508 | 0.985 |
| Firmicutes_Lachnospiraceae_unidentified_F23 | 1.30 | 1.02 | 0.985 | 0.985 |
| Firmicutes_Lachnospiraceae_unidentified_F16 | 1.55 | 0.69 | 0.508 | 0.985 |
| Firmicutes_Lachnospiraceae_unidentified_F18 | 0.91 | 0.97 | 0.835 | 0.985 |
| Firmicutes_Subdoligranulum_F30 | 0.48 | 1.30 | 0.984 | 0.985 |
| Bacteroidetes_Bacteroides_F29 | 0.94 | 0.55 | 0.145 | 0.985 |
| Firmicutes_Subdoligranulum_F25 | 0.78 | 0.42 | 0.832 | 0.985 |
| Firmicutes_Lactobacillus_F26 | 0.82 | 0.34 | 0.554 | 0.985 |
| Firmicutes_Ruminococcaceae_unidentified_F27 | 0.47 | 0.57 | 0.865 | 0.985 |
| Firmicutes_Subdoligranulum_F31 | 0.69 | 0.26 | 0.984 | 0.985 |
| Firmicutes_Clostridiales_unidentified_F41 | 0.50 | 0.46 | 0.417 | 0.985 |
| Firmicutes_Lachnospiraceae_unidentified_F33 | 0.64 | 0.26 | 0.002 * | 0.985 |
| Firmicutes_Lactobacillus_F22 | 0.46 | 0.40 | 0.891 | 0.985 |
| Firmicutes_Lachnospiraceae_incertae_sedis_F40 | 0.32 | 0.54 | 0.336 | 0.985 |

**Note**: Mean relative abundance (%) of the top 30 features are shown, with 15 high and 17 low RFI samples per intestinal segment. Statistical significance was determined using the Kruskal-Wallis test with Benjamini-Hochberg correction. False discovery rate (FDR) is shown.

**Figure S1. Multiple sequence alignment (A) and percent identity matrix (B) among six closely related Lachnospiraceae family members that are strongly associated with residual feed intake.**

**A**

F203 TGGGGAATATTGCACAATGGGGGAAACCCTGATGCAGCGACGCCGCGTGAAGGAAGAAGT

F76 TGGGGAATATTGCACAATGGGGGAAACCCTGATGCAGCGACGCCGCGTGAGCGAAGAAGT

F33 TGGGGAATATTGCACAATGGGGGAAACCCTGATGCAGCGACGCCGCGTGAGCGAAGAAGT

F92 TGGGGAATATTGCACAATGGGGGAAACCCTGATGCAGCGACGCCGCGTGAGCGAAGAAGT

F42 TGGGGAATATTGCACAATGGGGGAAACCCTGATGCAGCGACGCCGCGTGAAGGAAGAAGT

F116 TGGGGAATATTGCACAATGGGGGAAACCCTGATGCAGCGACGCCGCGTGAAGGAAGAAGT

************************************************** ********

F203 ATTTCGGTATGTAAACTTCTATCAGCAGGGAAGAAGAATGACGGTACCTGACTAAGAAGC

F76 ATCTCGGTATGTAAAGCTCTATCAGCAGGGAAGA-AAATGACGGTACCTGACTAAGAAGC

F33 ATTTCGGTATGTAAAGCTCTATCAGCAGGGAAGA-AACTGACGGTACCTGACTAAGAAGC

F92 ATTTCGGTATGTAAAGCTCTATCAGCAGGGAAGA-AACTGACGGTACCTGACTAAGAAGC

F42 ATCTCGGTATGTAAACTTCTATCAGCAGGGAAGA-AAGTGACGGTACCTGACTAAGAAGC

F116 ATTTCGGTATGTAAACTTCTATCAGCAGGGAAGA-AAATGACGGTACCTGACTAAGAAGC

** ************ ***************** * **********************

F203 CCCGGCTAACTACGTGCCAGCAGCCGCGGTAATACGTAGGGGGCAAGCGTTATCCGGATT

F76 ACCGGCTAAATACGTGCCAGCAGCCGCGGTAATACGTATGGTGCAAGCGTTATCCGGATT

F33 ACCGGCTAAATACGTGCCAGCAGCCGCGGTAATACGTATGGTGCAAGCGTTATCCGGATT

F92 ACCGGCTAAATACGTGCCAGCAGCCGCGGTAATACGTATGGTGCAAGCGTTATCCGGATT

F42 CCCGGCTAACTACGTGCCAGCAGCCGCGGTAATACGTAGGGGGCAAGCGTTATCCGGATT

F116 CCCGGCTAACTACGTGCCAGCAGCCGCGGTAATACGTAGGGGGCAAGCGTTATCCGGATT

******** **************************** ** ******************

F203 TACTGGGTGTAAAGGGAGCGTAGGCGGCCCGGCAAGTCAGAAGTGAAAACCCAGGGCTTA

F76 TACTGGGTGTAAAGGGAGCGTAGACGGAGAAGCAAGTCTGGAGTGAAAACCCGGGGCTCA

F33 TACTGGGTGTAAAGGGAGCGTAGACGGATTTGCAAGTCTGAAGTGAAAGCCCGGGGCTCA

F92 TACTGGGTGTAAAGGGAGCGTAGACGGATAGGCAAGTCTGGAGTGAAAGCCCGGGGCTCA

F42 TACTGGGTGTAAAGGGAGCGTAGACGGAATGGCAAGTCTGATGTGAAAGGCGGGGGCTCA

F116 TACTGGGTGTAAAGGGAGCGTAGACGGAGGAGCAAGTCCGATGTGAAACTCCGGGGCTTA

*********************** *** ******* * ****** * ***** *

F203 ACTCTGGGATTGCTTTTGAAACTGTCAGGCTCGATTGCCGGAGAGGTAAGTGGAATTCCT

F76 ACCCCGGGACTGCTTTGGAAACTGTTTTTCTGGAGTGCCGGAGAGGTAAGCGGAATTCCT

F33 ACCCCGGGACTGCTTTGGAAACTGTAGGTCTTGAGTGCTGGAGAGGTAAGTGGAATTCCT

F92 ACCCCGGGACTGCTTTGGAAACTGTTTATCTAGAGTGCTGGAGAGGCAAGTGGAATTCCT

F42 ACCCCTGGACTGCATTGGAAACTGCCAGTCTTGAGTACCGGAGGGGTAAGCGGAATTCCT

F116 ACCCCGGAACTGCATTGGAAACTGTTTATCTGGAGTGCCGGAGGGGTAAGCGGAATTCCT

** * * * *** ** ******* ** ** * * **** ** *** *********

F203 AGTGTAGCGGTGAAATGCGTAGATATTAGGAGGAACACCAGTGGCGAAGGCGGCTTACTG

F76 AGTGTAGCGGTGAAATGCGTAGATATTAGGAGGAACACCAGTGGCGAAGGCGGCTTACTG

F33 AGTGTAGCGGTGAAATGCGTAGATATTAGGAGGAACACCAGTGGCGAAGGCGGCTTACTG

F92 AGTGTAGCGGTGAAATGCGTAGATATTAGGAGGAACACCAGTGGCGAAGGCGGCTTGCTG

F42 AGTGTAGCGGTGAAATGCGTAGATATTAGGAGGAACACCAGTGGCGAAGGCGGCTTACTG

F116 GGTGTAGCGGTGAAATGCGTAGATATCAGGAGGAACACCGGTGGCGAAGGCGGCTTACTG

************************* ************ **************** ***

F203 GACGGTAAATGACGCTGAGGCTCGAAAGCGTGGGGAGCAAACA- 403

F76 GACGGTAACTGACGTTGAGGCTCGAAAGCGTGGGGAGCAAACAG 403

F33 GACAGTAACTGACGTTGAGGCTCGAAAGCGTGGGGAGCAAACAG 403

F92 GACAGTAACTGACGTTGAGGCTCGAAAGCGTGGGGAGCAAACAG 403

F42 GACGGTAACTGACGTTGAGGCTCGAAAGCGTGGGGAGCAAACAG 403

F116 GACGGTAACTGACGTTGAGGCTCGAAAGCGTGGGGAGCAAACAG 403

*** **** ***** ****************************

**B**

F203 F76 F33 F92 F42 F116

F203 100.00

F76 92.04 100.00

F33 92.04 96.53 100.00

F92 91.29 97.02 97.77 100.00

F42 92.79 93.55 93.80 92.56 100.00

F116 92.54 94.29 92.56 92.56 94.79 100.00
